# Supplementary material for: Serum/glucocorticoid-inducible kinase 1 deficiency induces NLRP3 inflammasome activation and autoinflammation of macrophages in a murine endolymphatic hydrops model
Source: Nat Commun. 2023 Mar 6;14:1249. doi: 10.1038/s41467-023-36949-4 (PMC9986248; doi:10.1038/s41467-023-36949-4)
Supplement: Supplementary file 1 — Supplementary information [file 41467_2023_36949_MOESM1_ESM.pdf]

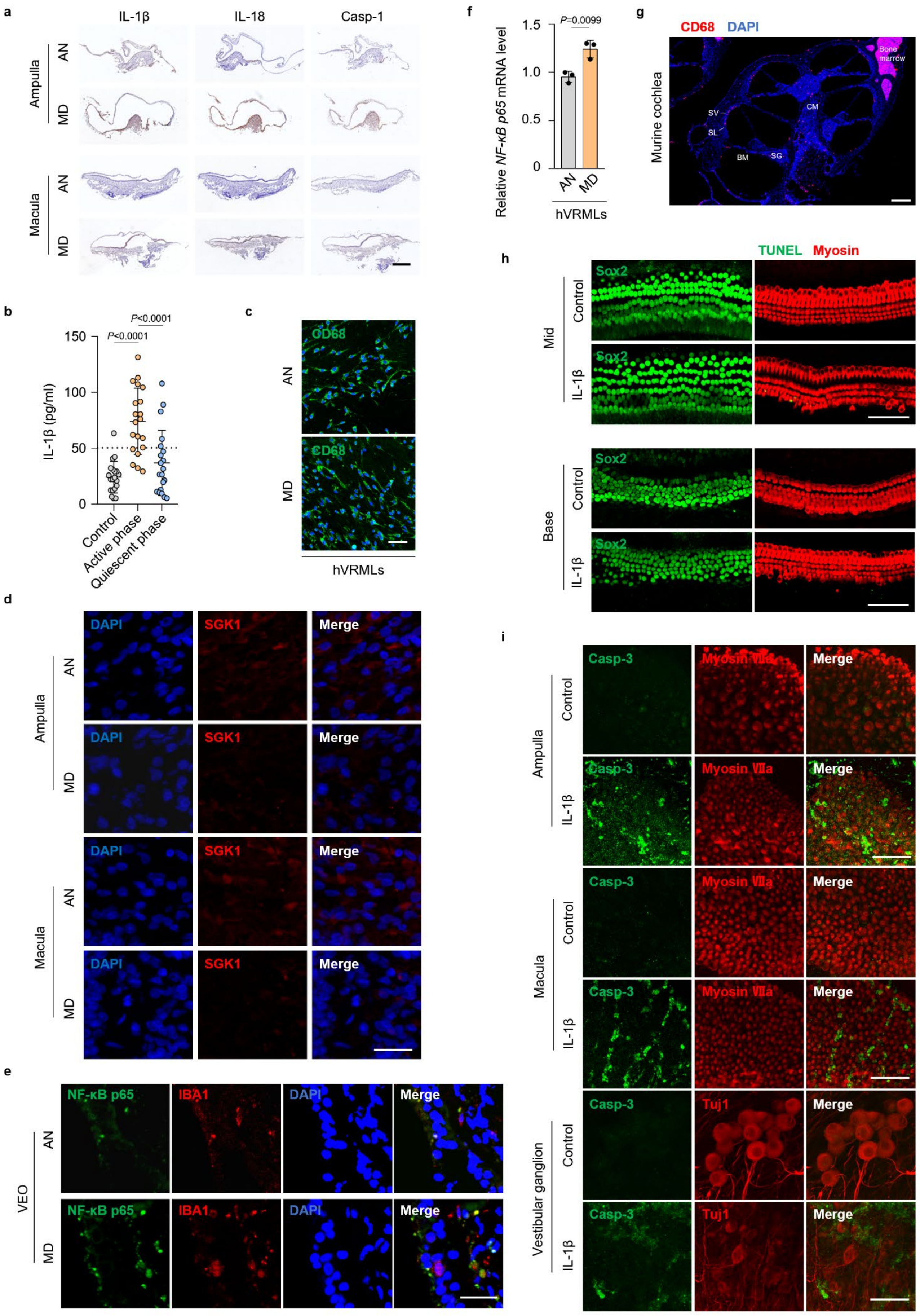

Supplementary Fig.1. a Representative images of immunohistochemical staining of IL-1β, IL-18, and Casp-1

in the ampulla and macula of AN (n=4) and MD patients (n=4), scale bar = 200 μm. **b** ELISA of plasma IL-1β levels in controls (n=20), MD patients during the active phase (n=20) and quiescent phase (n=20). The grid line represents the mean + 2 standard deviation of the controls. **c** Representative confocal microscopy images showing CD68 (green) and DAPI (blue) in hVRMLs cells of AN and MD patients, scale bar = 100 μm. **d** Representative confocal microscopy images showing SGK1 (red) and DAPI (blue) staining in the ampulla and macula of AN and MD patients (n=5), scale bar = 25μm. **e** Representative confocal microscopy images showing NF-κB p65 (green), IBA1 (red) and DAPI (blue) staining in the VEO of AN and MD patients (n=5), scale bar = 25 μm. **f** Quantitative real-time PCR analysis of *NF-κB p65* mRNA in hVRMLs of AN (n=3) and MD patients (n=3). **g** Representative confocal microscopy images showing CD68 (red) and DAPI (blue) staining of murine cochlea, scale bar = 200 μm. **h** Immunofluorescence staining for Sox2 (green, left), Myosin VIIa (red, right) and TUNEL (green, right) in the basilar membrane of middle and basal turn at P3, after treatment with IL-1β (10 ng/mL, 24 h) in vitro, scale bar = 50 μm. **i** Representative confocal microscopy images showing Myosin VIIa (red) and Casp-3 (green) staining in the ampulla and macula; Tuj1 (red) and Casp-3 (green) in the vestibular ganglion of mice, scale bar = 50 μm. Results are presented as mean ± SD. Statistical analyses were carried out via one-way ANOVA for **b** and two-sided t-test for **f**. Casp-1, caspase-1; Casp-3, caspase-3; hVRMLs, human vestibular-resident macrophage-like cells; VEO, vestibular end organs; CM, cochlear modiolus; SV, stria vascularis; SL, spiral ligament; SG, spiral ganglion; BM, basilar membrane.

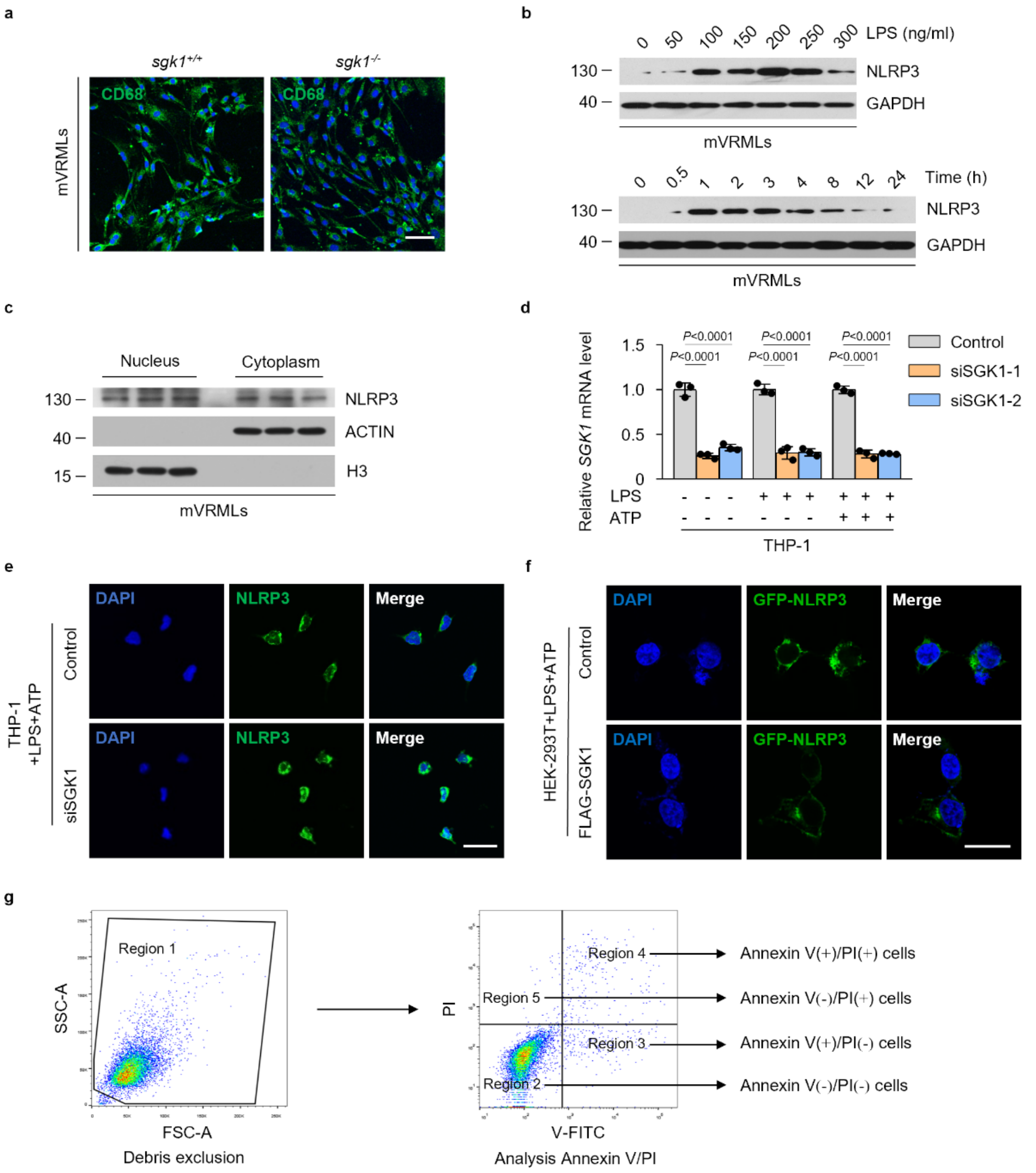

Supplementary Fig.2. a Representative immunofluorescence staining for CD68 (red) and DAPI (blue) in tissue-resident macrophage-like cells of *sgk1*$^{+/+}$ and *sgk1*$^{-/-}$ mice, scale bar = 100 μm. **b** Western blotting analysis was performed in mVRMLs upon stimulation with LPS of indicated concentration (up) and duration (down). **c** Western blotting analysis was performed in mVRMLs nucleus and cytoplasm. **d** Quantitative real-time PCR analysis of *SGK1* mRNA from control and SGK1 siRNA-transfected THP-1 cells (n=3 biologically independent experiments). **e** Representative confocal microscopy images showing NLRP3 (green) and DAPI (blue) in the control and SGK1 siRNA-transfected THP-1 cells that were stimulated with LPS (200 ng/mL, 3 h) and ATP (5 mM, 30 min), scale bar = 50 μm. **f** Representative confocal microscopy images showing NLRP3 (green) and DAPI (blue) in HEK-293T cells which overexpressed SGK1 and NLRP3 and stimulated with LPS

(200 ng/mL, 3 h) and ATP (5 mM, 30 min), scale bar = 25 μm. **g** Gating strategy for flow cytometry. Results are presented as mean ± SD. Statistical analyses were carried out via one-way ANOVA for **d**. mVRMLs, mouse vestibular-resident macrophage-like cells.

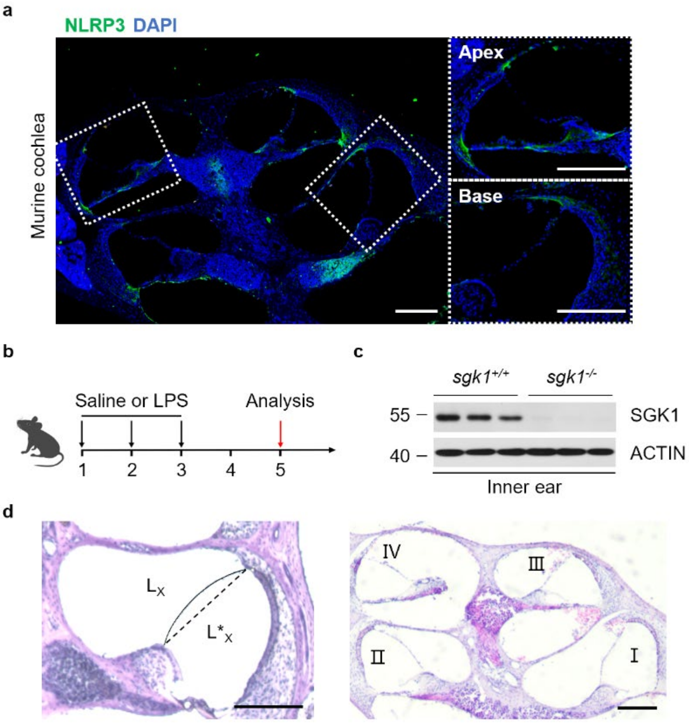

Supplementary Fig.3. a Representative confocal microscopy images showing NLRP3 (green) and DAPI (blue) staining in cochlea of the LPS-treated mice. The right panels represent 2-fold enlargements of the white dash lines areas in the left panels. scale bar = 200 μm. **b** Male C57BL/6 mice were treated with LPS (10 mg/kg, i.p., Sigma-Aldrich) or saline and sacrificed 5 days after the first injection. **c** Western blot analysis showing SGK1 level in *sgk1*$^{+/+}$ and *sgk1*$^{-/-}$ mice. **d** Representative images showing IR-L were measured in all four cochlea half turns, scale bar = 200 μm. IR-L, Increase ratios (IR) of the length of the Reissners membrane.

Supplementary table 1: Lists of chemicals and other resources.

REAGENT or RESOURCE	SOURCE	IDENTIFIER
Bacterial and virus strains		
BL21(DE3) competent cell	Vazyme	Cat# C504-02
DH5α Competent cell	Vazyme	Cat# C502-02
Biological samples		
Human peripheral blood	Shandong ENT hospital	XYK20190511
Human vestibular end organ	Shandong ENT hospital	XYK20190511
Chemicals, peptides, and recombinant proteins		
Ac-YVAD-cmk	InvivoGen	Cat# inh-yvad
Agarose	Solarbio	Cat# A8201
ATP	Sigma	Cat# FLAAS-5VL
β-mercaptoethanol	Procell	Cat# PB180633
Chemiluminescent HRP substrate	Millipore	Cat# WBKLS0500
DMSO	Abcam	Cat# ab120497
GSK650394	MCE	Cat# HY-15192
IL-1β	Shenandoah	Cat# 100--167AF
Kinase Assay buffer I	Abcam	Cat# ab189135
L-Glutathione reduced	Sigma	Cat# G4251
Lipfectamine RNAiMAX Reagent	Invitrogen	Cat# 13778-150
LPS	Sigma	Cat# L2880
Melanocyte medium	Melm	Cat# 2201
Melanocyte Growth Supplement	Melm	Cat# 2252
MCC950	AdipoGen	Cat# AG-CR1-2615-M010
Neutral balsam	Solarbio	Cat# G8590
Opti-MEM	Gibco	Cat# 31985-070
PEIpro	Polyplus Transfection	Cat# 115-0015
Phosphatase Inhibitor Cocktail I	MCE	Cat# HY-K0021
Phosphatase Inhibitor Cocktail II	MCE	Cat# HY-K0022
PMSF	Solarbio	Cat# P0100
ProLong Gold Antifade Mountant with DAPI	Invitrogen	Cat# P36935
Protease inhibitor cocktail	Sigma	Cat# P8340
RIPA lysis buffer	Beyotime Institute of Biotechnology	Cat# P0013B
Z-VAD FMK	Beyotime	Cat# C1202
Critical commercial assays		
Annexin V-FITC Apoptosis Detection Kit	Beyotime	Cat# C1062S
BCA protein Assary kit	Beyotime	Cat# P0012
DAB Detection Kit	ZSGB-BIO	Cat# SP9000
Human caspase-1 ELISA kit	Abcam	Cat# ab219633
Human IL-18 ELISA kit	Abcam	Cat# ab215539
Human IL-1β ELISA kit	RD	Cat# DLB50
Mouse caspase-1 ELISA kit	Novus	Cat# NBP2-75014
Mouse IL-1β ELISA kit	Abcam	Cat# ab197742
RevertAid Fist Strand cDNA Synthesis Kit	Thermo scientific	Cat# K1622
TB Green Premix EX Taq	TakaRa	Cat# RR420A
The TUNEL kit	Invitrogen	Cat# C10617

Continued		
Critical commercial assays		
Nuclear and Cytoplasmic Protein Extraction Kit	Beyotime	Cat# P0028
Experimental models: Cell lines		
HEK-293T	Procell	Cat# CL-0005
THP-1	Procell	Cat# CL-0233
Vestibular-resident macrophage-like cells	Neng et al., 2013	N/A
Experimental models: Organisms/strains		
Mouse: C57BL/6	Shandong university	C57BL/6J
Mouse: C57BL/6-Sgk1^{tm1cyagen}	Cyagen Biosciences	C57BL/6
Recombinant DNA		
EGFP-NLRP3	Addgene Plasmid	Cat# 73955; RRID:Addgene_73955
EGFP-NLRP3 LRR	This manuscript	N/A
EGFP-NLRP3 NACHT	This manuscript	N/A
EGFP-NLRP3 PYPIN	This manuscript	N/A
EGFP-NLRP3 ΔLRR	This manuscript	N/A
EGFP-NLRP3 ΔPYPIN	This manuscript	N/A
FLAG-SGK1	Oirgene	Cat# RC201535
FLAG-SGK1-S422A	This manuscript	N/A
FLAG-SGK1-S422D	This manuscript	N/A
GST-NLRP3	This manuscript	N/A
GST-NLRP3-S5A	This manuscript	N/A
Myc-ASC	Addgene Plasmid	Cat# 73952; RRID:Addgene_73952
Software and algorithms		
BioSigRZ-ABR	TDT	https://www.tdt.com/component/biosigrz-abr-dpoae-software/
FlowJo	FlowJo, LLC	https://www.flowjo.com/
GraphPad Prism	GraphPad Software	https://www.graphpad.com/
ImageJ	NIH	https://imagej.nih.gov/
Leica LAS AF	Leica Microsystem	https://leica-las-af-lite.software.informer.com/4.0/
CellSens Standard software	Olympus	https://www.olympus-lifescience.com.cn/en/software/cellsens/
Neuro-Audio	Neurosoft	https://neurosoft.com/en/catalog/audio/neuro-audio
BD FACSDiva Software	BD Bioscience	https://www.bdbiosciences.com/zh-cn/products/software/instrument-software/bd-facsdiva-software
SPSS	SPSS Inc	N/A
Other		
Anti-FLAG® M2 Magnetic Beads	Millipore	Cat# M8823; RRID:AB_2637089
Dynabeads™ Protein G	Invitrogen	Cat# 10004D
Glutathione Sepharose 4B beads	GE	Cat# 17075601
Polyvinylidene difluoride membranes	Millipore	Cat# ISEQ00010

Supplementary table 2. Clinical features of patients with MD.

Variables	Active phase (n=20)	Quiescent phase (n=20)
Age, Mean (SD)	60.9 (10.7)	58.9 (10.2)
Gender (%women)	11 (55%)	13 (65%)
Age of onset (SD)	51.3 (12.9)	52.0 (11.2)
Time course (years), mean (SD)	9.6 (16.7)	6.7 (5.1)
Side, n (%)		
Unilateral	17 (85%)	19 (95%)
bilateral	3 (15%)	1 (5%)
Clinical subtype, n (%)		
UMD 1, classical MD	11 (55%)	13 (65%)
UMD 2, delayed MD	1 (5%)	0 (0%)
UMD 4, migraine	3 (15%)	5 (25%)
UMD 5, autoimmune disease	2 (10%)	1 (5%)
BMD 1, metachronic sensorineural hearing loss	1 (5%)	1 (5%)
BMD 2, synchronic sensorineural hearing loss	1 (5%)	0 (0%)
BMD 4, migraine	1 (5%)	0 (0%)
Hearing loss at diagnosis, mean (SD)	72.5 (33.0)	65.5 (28.9)
Hearing stage, n (%)		
1	1 (5%)	2 (10%)
2	2 (10%)	2 (10%)
3	9 (45%)	10 (50%)
4	8 (40%)	6 (30%)
Tumarkin crisis, n (%)	2 (10%)	2 (10%)
High blood pressure, n (%)	5 (25%)	9 (45%)
Type 2 diabetes, n (%)	4 (20%)	1 (5%)
Autoimmune disease, n (%)	2 (10%)	1 (5%)
Migraine, n (%)	4 (20%)	5 (25%)
Functional level, n%		
1	0 (0%)	0 (0%)
2	1 (5%)	3 (15%)
3	5 (25%)	6 (30%)
4	2 (10%)	7 (35%)
5	8 (40%)	4 (20%)
6	4 (20%)	0 (0%)

Supplementary table 3: Lists of antibodies for immunostainings.

REAGENT or RESOURCE	SOURCE	IDENTIFIER	DILUTION
Alexa Fluor 488 donkey anti-goat IgG（H+L）	Invitrogen	Cat# A11055; RRID:AB_2534102	1:1000
Alexa Fluor 488 donkey anti-mouse IgG（H+L）	Invitrogen	Cat# A21202; RRID:AB_141607	1:1000
Alexa Fluor 488 donkey anti-rabbit IgG（H+L）	Invitrogen	Cat# A21206; RRID:AB_2535792	1:1000
Alexa Fluor 546 donkey anti-goat IgG（H+L）	Invitrogen	Cat# A11056; RRID:AB_2534103	1:1000
Alexa Fluor 546 donkey anti-mouse IgG（H+L）	Invitrogen	Cat# A10036; RRID:AB_2534012	1:1000
Alexa Fluor 546 donkey anti-rabbit IgG（H+L）	Invitrogen	Cat# A10040; RRID:AB_2534016	1:1000
Alexa Fluor 647 donkey anti-goat IgG（H+L）	Invitrogen	Cat# A21447; RRID:AB_2535864	1:1000
Alexa Fluor 647 donkey anti-mouse IgG（H+L）	Invitrogen	Cat# A21235; RRID:AB_2535804	1:1000
Alexa Fluor 647 donkey anti-rabbit IgG（H+L）	Invitrogen	Cat# A31573; RRID:AB_2536183	1:1000
Myosin VIIa	DSHB	Cat# 138-1-C	1:100
Caspase-1	Adipogen	Cat# AG-20B-0048B-C100; RRID:AB_2490257	1:200
Caspase-3	CST	Cat# 9664S; RRID:AB_2070042	1:200
CD68	Abcam	Cat# ab53444; RRID: AB_869007	1:200
FLAG	CST	Cat# 14793S; RRID:AB_2572291	1:200
GFP	Santa Cruz	Cat# sc-9996; RRID:AB_627695	1:200
IBA1	Abcam	Cat# ab5076; RRID:AB_91676	1:200
IL-18	Abcam	Cat# ab191152; RRID: AB_2737346	1:200
IL-1β	CST	Cat# 12242S; RRID:AB_2715503	1:200
MYC	CST	Cat# 2276; RRID:AB_331783	1:200
NK-κB p65	CST	Cat# 8242; RRID:AB_10859369	1:400
NLRP3	Invitrogen	Cat# MA5-23919; RRID: AB_2605972	1:200
SGK1	ABGENT	Cat# AP3924A-ev;	1:200
Sox2	Invitrogen	Cat# 14-9811-82; RRID: AB_11219471	1:200
Tuj1	Abcam	Cat# ab78078; RRID: AB_2792380	1:200

Supplementary table 4. RT-PCR Primers and siRNA

Oligonucleotides	SOURCE	IDENTIFIER
Human CASP1 Forward primer 5'-TTTCCGCAAGGTTCGATTTTCA-3'	This manuscript	N/A
Human CASP1 Reverse primer 5'-GGCATCTGCGCTCTACCATC-3'	This manuscript	N/A
Human IL18 Forward primer 5'-TCTTCATTGACCAAGGAAATCGG-3'	This manuscript	N/A
Human IL18 Reverse primer 5'-TCCGGGGTGCATTATCTCTAC-3'	This manuscript	N/A
Human IL1B beta Forward primer 5'-ATGATGGCTTATTACAGTGGCAA-3'	This manuscript	N/A
Human IL1B beta Reverse primer 5'-GTCGGAGATTCGTAGCTGGA-3'	This manuscript	N/A
Human NF-κB p65 Forward primer 5'-GTGGGGACTACGACCTGAATG-3'	This manuscript	N/A
Human NF-κB p65 Reverse primer 5'-GGGGCACGATTGTCAAAGATG-3'	This manuscript	N/A
Human NLRP3 Forward primer 5'-GATCTTCGCTGCGATCAACAG-3'	This manuscript	N/A
Human NLRP3 Reverse primer 5'-CGTGCATTATCTGAACCCCAC-3'	This manuscript	N/A
Human SGK1 Forward primer 5'-AGGATGGGTCTGAACGACTTT-3'	This manuscript	N/A
Human SGK1 Reverse primer 5'-GCCCTTTCCGATCACTTTCAAG-3'	This manuscript	N/A
Mouse CASP1 Forward primer 5'-ACAAGGCACGGGACCTATG-3'	This manuscript	N/A
Mouse CASP1 Reverse primer 5'-TCCCAGTCAGTCCTGGAAATG-3'	This manuscript	N/A
Mouse IL1B beta Forward primer 5'-GAAATGCCACCTTTTGACAGTG-3'	This manuscript	N/A
Mouse IL1B beta Reverse primer 5'-TGGATGCTCTCATCAGGACAG-3'	This manuscript	N/A
Mouse NLRP3 Forward primer 5'-ATTACCCGCCCGAGAAAGG-3'	This manuscript	N/A
Mouse NLRP3 Reverse primer 5'-CATGAGTGTGGCTAGATCCAAG-3'	This manuscript	N/A
siSGK1-1 CAGCUGAAAUGUACGACAAdTdT	This manuscript	N/A
siSGK1-2 GGCUACCUGCAUUCACUGAdTdT	This manuscript	N/A

Supplementary table 5: Lists of antibodies for immunoblot analysis.

REAGENT or RESOURCE	SOURCE	IDENTIFIER	DILUTION
ASC	Adipogen	Cat# AG-25B-0006-C100; RRID:AB_2885200	1:1000
Caspase-1	Adipogen	Cat# AG-20B-0042-C100; RRID:AB_2755041	1:1000
Caspase-1	Adipogen	Cat# AG-20B-0048B-C100; RRID:AB_2490257	1:1000
FLAG	CST	Cat# 14793S; RRID:AB_2572291	1:2000
GAPDH	Abcam	Cat# ab8245; RRID:AB_2107448	1:10000
Gasdermin D	CST	Cat# 96458S; RRID:AB_2894914	1:2000
GFP	Santa Cruz	Cat# sc-9996; RRID:AB_627695	1:2000
GST	CST	Cat# 2625S; RRID:AB_490796	1:1000
Histone H3	CST	Cat# 4499S; RRID: AB_10544537	1:2000
IL-1β	CST	Cat# 12242S; RRID:AB_2715503	1:1000
IPKine™ HRP, Goat Anti-Mouse IgG LCS	Abbkine	Cat# A25012; RRID:AB_2737290	1:10000
IPKine™ HRP, Mouse Anti-Rabbit IgG LCS	Abbkine	Cat# A25022; RRID:AB_2893334	1:10000
MYC	CST	Cat# 2276; RRID:AB_331783	1:2000
NLRP3	Invitrogen	Cat# MA5-23919; RRID: AB_2605972	1:2000
Normal Mouse IgG	Santa Cruz	Cat# sc-2025; RRID:AB_737182	1:2000
Normal Rabbit IgG	CST	C# 2729S; RRID:AB_1031062	1:2000
PARP1	CST	Cat# 9532S; RRID:AB_659884	1:1000
Peroxidase AffiniPure Goat Anti-Goat IgG (H+L)	Jackson	Cat# 705-035-003; RRID:AB_2340390	1:10000
Peroxidase AffiniPure Goat Anti-Mouse IgG (H+L)	Jackson	Cat# 115-035-003; RID:AB_10015289	1:10000
Peroxidase AffiniPure Goat Anti-Rabbit IgG (H+L)	Jackson	Cat# 111-035-003; RRID:AB_2313567	1:10000
Phosphoserine	Abcam	Cat# ab9332; RRID:AB_307184)	1:1000
SGK1	Invitrogen	Cat# PA5-85237; RRID: AB_2792380	1:1000
β-actin	CST	Cat# 3700S; RRID:AB_2242334	1:10000
