# Supplementary material for: Serum/glucocorticoid-inducible kinase 1 deficiency induces NLRP3 inflammasome activation and autoinflammation of macrophages in a murine endolymphatic hydrops model
Source: Nat Commun. 2023 Mar 6;14:1249. doi: 10.1038/s41467-023-36949-4 (PMC9986248; doi:10.1038/s41467-023-36949-4)
Supplement: Supplementary file 3 — Description of Additional Supplementary Files [file 41467_2023_36949_MOESM3_ESM.pdf]

Description of Additional Supplementary Files

File name: Supplementary Movie 1
Description: LPS or saline treated *sgk1*$^{+/+}$ and *sgk1*$^{-/-}$ mice are placed on a gradually accelerated motorized rotating rod.

File name: Supplementary Movie 2
Description: LPS and MCC950/saline treated *sgk1*$^{+/+}$ and *sgk1*$^{-/-}$ mice are placed on a gradually accelerated motorized rotating rod.
