# Supplementary material for: Serum/glucocorticoid-inducible kinase 1 deficiency induces NLRP3 inflammasome activation and autoinflammation of macrophages in a murine endolymphatic hydrops model
Source: Nat Commun. 2023 Mar 6;14:1249. doi: 10.1038/s41467-023-36949-4 (PMC9986248; doi:10.1038/s41467-023-36949-4)
Supplement: Supplementary file 6 — Reporting Summary [file 41467_2023_36949_MOESM6_ESM.pdf]

nature portfolio

Corresponding author(s): Hai-Bo Wang, Na Li

Last updated by author(s): Feb 8, 2023

Reporting Summary

Nature Portfolio wishes to improve the reproducibility of the work that we publish. This form provides structure for consistency and transparency in reporting. For further information on Nature Portfolio policies, see our Editorial Policies and the Editorial Policy Checklist.

Statistics

For all statistical analyses, confirm that the following items are present in the figure legend, table legend, main text, or Methods section.

n/a	Confirmed	
☐	☒	The exact sample size (n) for each experimental group/condition, given as a discrete number and unit of measurement
☐	☒	A statement on whether measurements were taken from distinct samples or whether the same sample was measured repeatedly
☐	☒	The statistical test(s) used AND whether they are one- or two-sided<br>*Only common tests should be described solely by name; describe more complex techniques in the Methods section.*
☒	☐	A description of all covariates tested
☒	☐	A description of any assumptions or corrections, such as tests of normality and adjustment for multiple comparisons
☐	☒	A full description of the statistical parameters including central tendency (e.g. means) or other basic estimates (e.g. regression coefficient) AND variation (e.g. standard deviation) or associated estimates of uncertainty (e.g. confidence intervals)
☐	☒	For null hypothesis testing, the test statistic (e.g. F, t, r) with confidence intervals, effect sizes, degrees of freedom and P value noted<br>*Give P values as exact values whenever suitable.*
☒	☐	For Bayesian analysis, information on the choice of priors and Markov chain Monte Carlo settings
☒	☐	For hierarchical and complex designs, identification of the appropriate level for tests and full reporting of outcomes
☒	☐	Estimates of effect sizes (e.g. Cohen's d, Pearson's r), indicating how they were calculated

Our web collection on statistics for biologists contains articles on many of the points above.

Software and code

Policy information about availability of computer code

Data collection	BioSigRZ-ABR software; BD FACSDiva Software version 9; Leica LAS AF; Neuro-Audio software; cellSens Standard software.
Data analysis	SPSS statistics 13.0; ImageJ; Graphpad prism 9; FlowJo, LLC.

For manuscripts utilizing custom algorithms or software that are central to the research but not yet described in published literature, software must be made available to editors and reviewers. We strongly encourage code deposition in a community repository (e.g. GitHub). See the Nature Portfolio guidelines for submitting code & software for further information.

Data

Policy information about availability of data

All manuscripts must include a data availability statement. This statement should provide the following information, where applicable:

- Accession codes, unique identifiers, or web links for publicly available datasets
- A description of any restrictions on data availability
- For clinical datasets or third party data, please ensure that the statement adheres to our policy

All data generated or analyzed during this study are present in the main text and supplementary data. Origin data of all figures presented in the main text and supplementary data are provided in source data file.

Human research participants

Policy information about studies involving human research participants and Sex and Gender in Research.

Reporting on sex and gender	The term sex were used in this manuscript and determined based on medical record. The female ratio is shown in supplementary table 1.
Population characteristics	ALL patients have been diagnosed with MD, aged 34–87 years (average age 59.9 years). Population characteristics are shown in the methods section and supplementary table 1.
Recruitment	ALL patients were diagnosed with MD and recruited after informed consent was obtained. No bias on selecting patients existed.
Ethics oversight	This protocol was reviewed by the ethic committee of Shandong Provincial ENT Hospital affiliated to Shandong University and according to the principles of the Declaration of Helsinki revised in 2013 for investigation with humans. Informed consent was obtained from all participants.

Note that full information on the approval of the study protocol must also be provided in the manuscript.

Field-specific reporting

Please select the one below that is the best fit for your research. If you are not sure, read the appropriate sections before making your selection.

[x] Life sciences [ ] Behavioural & social sciences [ ] Ecological, evolutionary & environmental sciences

For a reference copy of the document with all sections, see nature.com/documents/nr-reporting-summary-flat.pdf

Life sciences study design

All studies must disclose on these points even when the disclosure is negative.

Sample size	No statistical methods were used to predetermined sample size. Sample sizes were based on preliminary experiments. Animal sample using at least 3 animals per group. Experimental approach, availability and feasibility required to obtain statistically significant results were also taken in consideration. The sample size was at least 3 for analysis as stated in the figure legends.
Data exclusions	No samples were excluded from analysis.
Replication	The number of independent samples of each experiment can be found in the relevant figure legend. For western blots, qPCR, flow cytometry and other quantitative experiment were performed at least three times. For IF/IHC/HE stains, the number of independent samples is consistent with the relevant quantitative graph, the images are representative images of at least three independent replicates of the experiments.
Randomization	In all animal studies, mice were grouped by genotype and matched by age and sex. No methods of randomization was used. No randomization was done for the analyses of human biopsy material as this involved no intervention.
Blinding	The investigators were blinded to group allocation during the pathology and function analysis, and data analysis.

Reporting for specific materials, systems and methods

We require information from authors about some types of materials, experimental systems and methods used in many studies. Here, indicate whether each material, system or method listed is relevant to your study. If you are not sure if a list item applies to your research, read the appropriate section before selecting a response.

Materials & experimental systems

n/a	Involved in the study	
[ ]	[x]	Antibodies
[ ]	[x]	Eukaryotic cell lines
[x]	[ ]	Palaeontology and archaeology
[ ]	[x]	Animals and other organisms
[x]	[ ]	Clinical data
[x]	[ ]	Dual use research of concern

Methods

n/a	Involved in the study	
[x]	[ ]	ChIP-seq
[ ]	[x]	Flow cytometry
[x]	[ ]	MRI-based neuroimaging

Antibodies

Antibodies used

Western Blot: Anti-NLRP3 (768319, Invitrogen, MA5-23919, dilution 1:2000); Anti-SGK1 (Polyclonal, Invitrogen, PA5-85237, dilution 1:1000); Anti-GAPDH (6C5, Abcam, ab8245, dilution 1:10000); Anti-Caspase-1 (Casper-1, Adipogen, AG-20B-0042-C100, dilution 1:1000); Anti-Caspase-1 (Bally-1, Adipogen, AG-20B-0048B-C100, dilution 1:1000); Anti-IL-1β (3A6, CST, 12242S, dilution 1:1000); Anti-Gasdermin D (Polyclonal, CST, 96458S, dilution 1:2000); Anti-PARP1 (46D11, CST, 9532S, dilution 1:1000); Anti-GFP (B-2, Santa Cruz, sc-9996, dilution 1:2000); Anti-Phosphoserine (Polyclonal, Abcam, ab9332, dilution 1:1000); Anti-GST (91G1, CST, 2625S, dilution 1:1000); Anti-ASC (Polyclonal, Adipogen, AG-25B-0006-C100, dilution 1:1000); Anti-FLAG (D6W5B, CST, 14793S, dilution 1:2000); Anti-MYC (9B11, CST, 2276, dilution 1:2000); Anti-Normal mouse IgG (Santa Cruz, sc-2025, dilution 1:2000); Anti-Normal Rabbit IgG (CST, 2729S, dilution 1:2000); Anti-Histone H3 (D1H2, CST, 4499S, dilution 1:2000); Anti-β-actin (8H10D10, CST, 3700S, dilution 1:10000); IPKine™ HRP, Goat Anti-Mouse IgG LCS (Abbkine, A25012, dilution 1:10000); IPKine™ HRP, Mouse Anti-Rabbit IgG LCS (Abbkine, A25022, dilution 1:10000); Peroxidase AffiniPure Goat Anti-Goat IgG (H+L) (Jackson, 705-035-003, dilution 1:10000); Peroxidase AffiniPure Goat Anti-Mouse IgG (H+L) (Jackson, 115-035-003, dilution 1:10000); Peroxidase AffiniPure Goat Anti-Rabbit IgG (H+L) (Jackson, 111-035-003, dilution 1:10000).

Immunofluorescence and immunohistochemistry: Anti-IBA1 (Polyclonal, Abcam, ab5076, dilution 1:200); Anti-IL-1β (3A6, CST, 12242S, dilution 1:200); Anti-Caspase-1 (Bally-1, Adipogen, AG-20B-0048B-C100, dilution 1:200); Anti-IL-18 (Polyclonal, Abcam, ab191152, dilution 1:200); Anti-Sox2 (Btjce, Invitrogen, 14-9811-82, dilution 1:200); Anti-Tuj1 (2G10, Abcam, ab78078, dilution 1:200); Anti- Myosin Ⅶa (927-1203, DSHB, 138-1-C, dilution 1:100); Anti-SGK1 (Ser78, ABGENT, AP3924A-ev, dilution 1:2000); Anti-CD68 (FA-11, Abcam, ab53444, dilution 1:200); Anti-Caspase-3 (5A1, CST, 9664S, dilution 1:200); Anti-NLRP3 (768319, Invitrogen, MA5-23919, dilution 1:200); Anti-GFP (B-2, Santa Cruz, sc-9996, dilution 1:200); Anti-MYC (9B11, CST, 2276, dilution 1:200); Anti-FLAG (D6W5B, CST, 14793S, dilution 1:200); Anti-NF-kB p65 (D14E12, CST, 8242, dilution 1:400) ; Alexa Fluor 488 donkey anti-goat IgG (H+L) (Invitrogen, A11055, dilution 1:1000); Alexa Fluor 488 donkey anti-mouse IgG (H+L) (Invitrogen, A21202, dilution 1:1000); Alexa Fluor 488 donkey anti-rabbit IgG (H+L) (Invitrogen, A21206, dilution 1:1000); Alexa Fluor 546 donkey anti-goat IgG (H+L) (Invitrogen, A11056, dilution 1:1000); Alexa Fluor 546 donkey anti-mouse IgG (H+L) (Invitrogen, A10036, dilution 1:1000); Alexa Fluor 546 donkey anti-rabbit IgG (H+L) (Invitrogen, A10040, dilution 1:1000); Alexa Fluor 647 donkey anti-goat IgG (H+L) (Invitrogen, A21447, dilution 1:1000); Alexa Fluor 647 donkey anti-mouse IgG (H+L) (Invitrogen, A21235, dilution 1:1000); Alexa Fluor 647 donkey anti-rabbit IgG (H+L) (Invitrogen, A31573, dilution 1:1000).

Validation

All antibodies listed above were commercially available and validated by the manufacturer and were previously used in other studies. Validation was available from the websites of respective vendors:
Anti-NLRP3: https://www.thermofisher.cn/cn/zh/antibody/product/Donkey-anti-Goat-IgG-H-L-Cross-Adsorbed-Secondary-Antibody-Polyclonal/A-11055
Anti-SGK1: https://www.thermofisher.cn/cn/zh/antibody/product/SGK1-Antibody-Polyclonal/PA5-85237
Anti-GAPDH: https://www.abcam.cn/gapdh-antibody-6c5-loading-control-ab8245.html
Anti-Caspase-1: https://adipogen.com/storeconfig/choose/store?destination: ag-20b-0042-anti-caspase-1-p20-mouse-mab-casper-1.html
Anti-Caspase-1: https://adipogen.com/storeconfig/choose/store?destination: ag-20b-0048b-anti-caspase-1-p20-human-mab-bally-1-biotin.html
Anti-IL-1β: https://www.cellsignal.cn/products/primary-antibodies/il-1b-3a6-mouse-mab/12242
Anti-Gasdermin D: https://www.cellsignal.cn/products/primary-antibodies/gasdermin-d-antibody/96458
Anti-PARP1: https://www.cellsignal.cn/products/primary-antibodies/parp-46d11-rabbit-mab/9532
Anti-GFP: https://www.scbt.com/zh/p/gfp-antibody-b-2/
Anti-Phosphoserine: https://www.abcam.cn/phosphoserine-antibody-ab9332.html
Anti-GST: https://www.cellsignal.com/products/primary-antibodies/gst-91g1-rabbit-mab/2625
Anti-ASC: https://adipogen.com/storeconfig/choose/store?destination: ag-25b-0006-anti-asc-pab-al177.html
Anti-FLAG: https://www.cellsignal.cn/products/primary-antibodies/dykddddk-tag-d6w5b-rabbit-mab-binds-to-same-epitope-as-sigma-s-anti-flag-m2-antibody/14793
Anti-MYC: https://www.cellsignal.com/products/primary-antibodies/myc-tag-9b11-mouse-mab/2276
Anti-Normal mouse IgG: https://www.scbt.com/zh/p/normal-mouse-igg/
Anti-Normal Rabbit IgG: https://www.cellsignal.cn/products/primary-antibodies/normal-rabbit-igg/2729
Anti-Histone H3: https://www.cellsignal.cn/products/primary-antibodies/histone-h3-d1h2-xp-rabbit-mab/4499
Anti-β-actin: https://www.cellsignal.cn/products/primary-antibodies/b-actin-8h10d10-mouse-mab/3700
IPKine™ HRP, Goat Anti-Mouse IgG LCS: https://www.abbkine.cn/product/a25012/
IPKine™ HRP, Mouse Anti-Rabbit IgG LCS: https://www.abbkine.com/product/ipkine-hrp-mouse-anti-rabbit-igg-lcs-a25022/
Peroxidase AffiniPure Goat Anti-Goat IgG (H+L): https://www.jacksonimmuno.com/catalog/products/705-035-003
Peroxidase AffiniPure Goat Anti-Mouse IgG (H+L): https://www.jacksonimmuno.com/catalog/products/115-035-003
Peroxidase AffiniPure Goat Anti-Rabbit IgG (H+L): https://www.jacksonimmuno.com/catalog/products/111-035-003
Anti-IBA1: https://www.abcam.cn/Iba1-antibody-ab5076.html
Anti-Caspase-1: https://adipogen.com/storeconfig/choose/store?destination: ag-20b-0048b-anti-caspase-1-p20-human-mab-bally-1-biotin.html
Anti-IL-18: https://www.abcam.cn/il-18-antibody-ab191152.html
Anti-Sox2: https://www.thermofisher.cn/cn/zh/antibody/product/SOX2-Antibody-clone-Btjce-Monoclonal/14-9811-82
Anti-Tuj1: https://www.abcam.cn/beta-iii-tubulin-antibody-2g10-neuronal-marker-ab78078.html
Anti- Myosin Ⅶa: https://dshb.biology.uiowa.edu/MYO7A-138-1
Anti-SGK1: https://www.abcepta.com.cn/products/AP3924a-SGK-Ser78-Antibody
Anti-CD68: https://www.abcam.cn/cd68-antibody-fa-11-ab53444.html
Anti-Caspase-3: https://www.cellsignal.cn/products/primary-antibodies/cleaved-caspase-3-asp175-5a1e-rabbit-mab/9664
Anti-NLRP3: https://www.thermofisher.cn/cn/zh/antibody/product/NLRP3-Antibody-clone-768319-Monoclonal/MA5-23919
Anti-NF-kB p65: https://www.cellsignal.cn/products/primary-antibodies/nf-kb-p65-d14e12-xp-rabbit-mab/8242
Alexa Fluor 488 donkey anti-goat IgG (H+L): https://www.thermofisher.cn/cn/zh/antibody/product/Donkey-anti-Goat-IgG-H-L-Cross-

Adsorbed-Secondary-Antibody-Polyclonal/A-11055
Alexa Fluor 488 donkey anti-mouse IgG (H+L): https://www.thermofisher.cn/cn/zh/antibody/product/Donkey-anti-Mouse-IgG-H-L-Highly-Cross-Adsorbed-Secondary-Antibody-Polyclonal/A-21202
Alexa Fluor 488 donkey anti-rabbit IgG (H+L): https://www.thermofisher.cn/cn/zh/antibody/product/Donkey-anti-Rabbit-IgG-H-L-Highly-Cross-Adsorbed-Secondary-Antibody-Polyclonal/A-21206
Alexa Fluor 546 donkey anti-goat IgG (H+L): https://www.thermofisher.cn/cn/zh/antibody/product/Donkey-anti-Goat-IgG-H-L-Cross-Adsorbed-Secondary-Antibody-Polyclonal/A-11056
Alexa Fluor 546 donkey anti-mouse IgG (H+L): https://www.thermofisher.cn/cn/zh/antibody/product/Donkey-anti-Mouse-IgG-H-L-Highly-Cross-Adsorbed-Secondary-Antibody-Polyclonal/A10036
Alexa Fluor 546 donkey anti-rabbit IgG (H+L): https://www.thermofisher.cn/cn/zh/antibody/product/Donkey-anti-Rabbit-IgG-H-L-Highly-Cross-Adsorbed-Secondary-Antibody-Polyclonal/A10040
Alexa Fluor 647 donkey anti-goat IgG (H+L): https://www.thermofisher.cn/cn/zh/antibody/product/Donkey-anti-Goat-IgG-H-L-Cross-Adsorbed-Secondary-Antibody-Polyclonal/A-21447
Alexa Fluor 647 donkey anti-mouse IgG (H+L): https://www.thermofisher.cn/cn/zh/antibody/product/Goat-anti-Mouse-IgG-H-L-Cross-Adsorbed-Secondary-Antibody-Polyclonal/A-21235
Alexa Fluor 647 donkey anti-rabbit IgG (H+L): https://www.thermofisher.cn/cn/zh/antibody/product/Donkey-anti-Rabbit-IgG-H-L-Highly-Cross-Adsorbed-Secondary-Antibody-Polyclonal/A-31573

Eukaryotic cell lines

Policy information about cell lines and Sex and Gender in Research

Cell line source(s)	HEK-293T and THP-1 were all purchased from Procell (Wuhan, China).
Authentication	The cell line was authenticated by the vendor. We did not perform any validation except for visual evaluation of morphology.
Mycoplasma contamination	Cells were routinely tested for mycoplasma, and we confirm that every cell used in this study was found negative.
Commonly misidentified lines (See ICLAC register)	No commonly misidentified cell lines were used in this study.

Animals and other research organisms

Policy information about studies involving animals; ARRIVE guidelines recommended for reporting animal research, and Sex and Gender in Research

Laboratory animals	Wild-type (WT) C57BL/6 (2-month-old male) mice were purchased from the Animal Center of Shandong University. Sgk1 knocked out (sgk1-/-, male, 2 months) mice were purchased form Cyagen Biosciences. Mice were housed in a temperature-controlled (20-22 ℃) room with 40–70% humidity, subjected to a 12/12 h light/dark cycle, and had free access to food and drinking water. Experiments were performed on age- and sex-matched 8- to 10-week-old mice weighing 17-25 g.
Wild animals	Not uesd.
Reporting on sex	Both male and female mice have been used in our current experiments.
Field-collected samples	Not uesd.
Ethics oversight	All study protocols were approved by the Animal Care Committee of Shandong University (Jinan, China) and conformed with the Guideline for the Care and Use of Laboratory Animal of the National institutes of Health.

Note that full information on the approval of the study protocol must also be provided in the manuscript.

Flow Cytometry

Plots

Confirm that:

- [x] The axis labels state the marker and fluorochrome used (e.g. CD4-FITC).
- [x] The axis scales are clearly visible. Include numbers along axes only for bottom left plot of group (a 'group' is an analysis of identical markers).
- [x] All plots are contour plots with outliers or pseudocolor plots.
- [x] A numerical value for number of cells or percentage (with statistics) is provided.

Methodology

Sample preparation	Cells were harvested, washed with PBS solution prior to staining.
Instrument	LSRII flow cytometer (BD Bioscience).

Software	BD FACSDiva Software version 9 (BD Bioscience); FlowJo, LLC.
Cell population abundance	Abundance and purity of cell population determined by FlowJo software with identical gating strategy across each set of samples.
Gating strategy	Cells were discriminated from debris and clumps using the FSC-A/SCC-A gating strategy based on experience. Only single cells were used using FSC-H/FSC-A gating strategy and selecting cells along the diagonal. The gating strategy is provided in Supplementary Fig. 2g.

☒ Tick this box to confirm that a figure exemplifying the gating strategy is provided in the Supplementary Information.
